# Fall prediction in a quiet standing balance test via machine learning: Is it possible?

**DOI:** 10.1371/journal.pone.0296355

**Published:** 2024-04-16

**Authors:** Juliana Pennone, Natasha Fioretto Aguero, Daniel Marczuk Martini, Luis Mochizuki, Alexandre Alarcon do Passo Suaide

**Affiliations:** 1 Department of Orthopedics and Traumatology, Hospital das Clínicas, Faculty of Medicine, University of São Paulo, São Paulo, Brazil; 2 School of Arts, Sciences and Humanities, University of Sao Paulo, São Paulo, Brazil; 3 Institute of Physics, University of São Paulo, São Paulo, Brazil; Universita Politecnica delle Marche Facolta di Ingegneria, ITALY

## Abstract

The elderly population is growing rapidly in the world and falls are becoming a big problem for society. Currently, clinical assessments of gait and posture include functional evaluations, objective, and subjective scales. They are considered the gold standard to indicate optimal mobility and performance individually, but their sensitivity and specificity are not good enough to predict who is at higher risk of falling. An innovative approach for fall prediction is the machine learning. Machine learning is a computer-science area that uses statistics and optimization methods in a large amount of data to make outcome predictions. Thus, to assess the performance of machine learning algorithms in classify participants by age, number of falls and falls frequency based on features extracted from a public database of stabilometric assessments. 163 participants (116 women and 47 men) between 18 and 85 years old, 44.0 to 75.9 kg mass, 140.0 to 189.8 cm tall, and 17.2 to 31.9 kg/m^2^ body mass index. Six different machine learning algorithms were tested for this classification, which included Logistic Regression, Linear Discriminant Analysis, K Nearest-neighbours, Decision Tree Classifier, Gaussian Naive Bayes and C-Support Vector Classification. The machine learning algorithms were applied in this database which has sociocultural, demographic, and health status information about participants. All algorithm models were able to classify the participants into young or old, but our main goal was not achieved, no model identified participants at high risk of falling. Our conclusion corroborates other works in the biomechanics field, arguing the static posturography, probably due to the low daily living activities specificity, does not have the desired effects in predicting the risk of falling. Further studies should focus on dynamic posturography to assess the risk of falls.

## Introduction

For the elderly, the world’s average prevalence of falls is 26.5% (95% CI 23.4–29.8%) [1]. As the elderly grows rapidly in the world [2], falling are becoming a huge social problem. Currently, the gait and posture clinical assessments include functional evaluations, objective and subjective scales. They are considered the gold standard for an optimal mobility and performance individually [3], but their sensitivity and specificity are not good to predict fall [4]. Although they are easily applied in clinical practice and considered the gold standard, they are not reliable for the daily living activities [4]. Novel approaches for fall prediction are emerging to account for real-time fall prediction and future fall prediction, which are necessary to improve health care systems, and both are based on the proper fall risk assessment [5, 6]. The prognosis and early selection of an intervention to reduce or to prepare the individuals for falls demands an efficient predictive tool to identify older adults at higher risk of falling [5, 7].

A novel approach for fall prediction is the machine learning (ML). ML is a computer-science field that uses statistics and optimization methods for a large amount of data to predict outcomes [8–10]. When fed with new data, ML focuses on classification, which involves choosing among subgroups to best describe a new data instance, and prediction, which involves estimating an unknown parameter. ML is more efficient in predictions than the traditional statistical analysis [11–13]. In medicine, health and wellness fields, ML has improved clinical-decision making, assisting in diagnosing Parkinson’s disease [14], breast masses [15] or even aiding in the classification of health-related quality of life for older adults with chronic disease [16].

ML can classify gait patterns. ML has been applied for classifying physical activities or movement patterns. It is efficient in separating young’ and older adults’ gait patterns [17], older adults who do or do not fall recurrently [18], detecting falls with different sensors and sensor locations [6, 19–23], predicting fall-related severe outcome and injury [24], and predicting the fallers mortality [25]. About stratifying the elderly at risk of falling, ML has been applied to medical records and organizational factors in nursing homes [26], patient demographics, historical visits, visit patterns and diagnoses data after emergency department visits [27], and gait spatial-temporal parameters [28–30]. ML has been also applied in static balance tests, but its efficacy in distinguishing individuals at higher fall risk is controversial and its value in predicting falls remains unclear [31–33].

Thus, the aim of this study was to analyze the stabilometric assessment of adult balance using ML to classify people with different annual frequencies of accidental falls in a public database related to human balance [34]. Six different ML algorithms were tested for this classification, which included Logistic Regression (LR), Linear Discriminant Analysis (LDA), K Nearest-neighbours (KNN), Decision Tree Classifier (CART), Gaussian Naive Bayes (NB) and C-Support Vector Classification (SVC). A comparison among their performances is also presented. To the best of our knowledge, this is the first study to apply ML in a static balance test into a greater age spectrum, which can mark clear differences among participants. Our hypothesis was that supervised ML would detect patterns in a static balance test that characterize non-fallers (who had never fallen one year before data collection) and fallers (who had fallen at least once up to one year before data collection).

## Method

This study used a public data repository, all participants (163, 116 women and 47 men), between 18 and 85 years old, were informed about the study’ purpose and procedures, and gave the free informed consent, which terms were approved by the UFABC ethics committee #842529/2014 [34]. The ML algorithm was applied in this database which has sociocultural, demographic and health status information about participants.

### Participants

The participants’ main characteristics were: 44.0 to 75.9 kg mass, 140.0 to 189.8 cm tall, and 17.2 to 31.9 kg/m^2^ body mass index. Ten percent of adults had one or more severe disabilities, including hearing, vestibular, visual, intelligence, and musculoskeletal deficits. For more information, refer to [34].

### Balance evaluation

Four experimental conditions were analyzed: standing on a hard surface with closed or open eyes, and standing on a foam surface with closed or open eyes open. All conditions were recorded for 60 s on a force platform (OPT400600-1000 model, AMTI, Watertown, MA, USA), feet with an angle of 20 degrees and 10 cm apart from heels. Ground reaction forces and moments of forces were recorded (100 Hz sampling frequency, Optima Signal Conditioner, AMTI, Watertown, MA, USA), low-pass filtered (10 Hz 4th order Butterworth filter) and used to calculate the center of pressure (COP). There were three trials in each condition for every individual.

### Analyzed parameters

Features from AP (*x*-positive is anterior) and medio-lateral ML (y-positive is to the right) COP were calculated) of all three subjects’ attempts in each type of test configuration (standing on a hard surface with eyes open, standing on a hard surface with eyes closed, standing on a foam surface with eyes open and standing on a foam surface with eyes closed):area, mean velocity, maximum excursion, and RMS value. Each question from Mini Balance Evaluation Systems Tests (Mini-BESTest), Short Falls Efficacy Scale International (Short FES-I), International Physical Activity Questionnaire—Short Version (IPAQ-SV) and Trail Making Test (TMT) was analyzed separately. Thus, IA models features were all 53 biomechanical parameters obtained from COP tests and demographic data: age, sex, height, weight and BMI.

In this work, the following six supervised learning models are used:

#### Logistic Regression (LR)

This method consists of applying statistical models to separate binary results, for example, if an email is SPAM or not. A relationship between the expected result and the independent variables is established by estimating probabilities through a cumulative logistic function. In this model, the probabilities that describe the possible outcomes of a single trial are modeled using a logistic function [35], such as the equation:

$$f(x)=\frac{L}{1+e^{-k(x-x0)}}$$

where x0 is the value of x at the midpoint of the sigmoid curve; L is the maximum value of the curve and k is the slope of the curve.

#### Linear Discriminant Analysis (LDA)

Linear Discriminant Analysis is a classifier that employs a linear decision surface. Among its advantages, LDA has closed format solutions that can be easily computed. Also, LDA is inherently multiclass and does not need to have adjustable hyperparameters [36].

#### K-nearest neighbors (KNN)

This is a non-parametric algorithm, that is, there is no assumption about the distribution of the input data. It is based on searching for similarities: the data points are classified using the distance between them, looking for their nearest k neighbors. Then, these data will be sorted to the most common class among these k-neighbors [37].

#### Decision Tree (CART)

Decision Trees are a non-parametric supervised learning method employed in classification and regression. The objective is to create a model that predicts the value of a variable by learning simple decision rules inferred from data parameters. A tree can be seen as a constant approximation of functions defined in chunks. The deeper the tree, the more complex the decision rules will be [38].

#### Gaussian Naive Bayes (NB)

Naive Bayes methods are a set of algorithms based on the application of Bayes’ theorem with the naive assumption of conditional independence between each pair of parameters [39]. The probability of resources is assumed to be Gaussian, as shown in the equation; y and y are estimated using the maximum likelihood [39]:

$$P\left(x_{i}\;|y\right)=\frac{1}{\sqrt{2\pi \sigma_{y}^{2}}}exp\left(-\frac{{\left(x_{i}-\mu_{y}\right)}^{2}}{2\sigma_{y}^{2}}\right)$$

#### C-Support Vector Classification (SVM)

Support vector machines (SVM) are a set of supervised learning methods used for classification, regression and also outlier detection. From a vector of input data, with N dimensions, (N-1) interface hyperplanes are generated. In general, these algorithms construct a decision line between the results in each of these hyperplanes and these interfaces are used, further on, to make predictions [40].

The performance of a ML program can be evaluated based on performance parameters linked to the classification capacity of the model, such as sensitivity, precision and accuracy.:

#### Precision

Refers to the repeatability of the results [40]. It is defined by dividing the number of true positives (TP) by the total number of positives found, TP+FP (FP stands for false positives):

$$Precision=\frac{TP}{TP+FP}$$

#### Sensitivity or recall

Refers to the model’s ability to avoid false negatives [40]. It is defined by dividing the number of true positives by the total number of elements that belong to the positive group, TP+FN:

$$Sensitivity=\frac{TP}{TP\;+FN}$$

#### Accuracy

Refers to how close a variable measure is to its actual value [40]. It is defined as the proportion of true results (true positives TP and true negatives TN) by total results:

$$Accuracy=\frac{TP\;+\;TN}{TP\;+TN+FP+FN}$$

Ideally, precision, sensitivity and accuracy should be equal to 1 (or 100%); however, values very close to 1 may indicate overfitting of the training data.

Santos & Duarte public database was analyzed with the before-mentioned supervised ML algorithms. The models were trained with all 53 biomechanical parameters obtained from the force balance. All data were normalized for the analysis methods used using z-scores with respect to their mean values. When missing data occurred, they were replaced with the mean of that specific attribute. For supervised algorithms, the analysis was carried out using data from week zero, while data from subsequent weeks were used for validation of this training to observe if there is a change in individual classification or any temporal trends. A code authored by Jason Brownlee [41] was adapted to analyze the performance of the different algorithms. The training performance of different algorithms was conducted by randomly dividing the data into two groups: 80% of the data were used for training, and the remaining 20% for validation tests. This was repeated at least 10 times in order to avoid accidental bias due to the splitting.

In the case of supervised learning, an a priori labeling of the training data must be used. To explore different prediction possibilities, three types of classification, as suggested by Santos and Duarte, 2016; were performed, each in a separate program:

- Age: Young: Age < 60 (88 young subjects); Old: Age ≥ 60 (75 elderly subjects)
- Falls: how many non-intentional falls the subject had in the last 12 months, as declared by themselves—No falls (zero falls– 120 subjects); One fall or more (from 0 to…– 43 subjects).
- Fall frequency: how many non-intentional falls the subject had in the last 12 months, as declared by themselves—Non-faller (from 0 to 1–128 subjects); Faller (from 1 to…– 35 subjects)

All code was developed in Python 3.7 language. The ML algorithms belong to the sklearn library and were implemented via the these functions: Logistic Regression, Linear Discriminant Analysis, Decision Tree Classifier, Gaussian NB, SVC, K-Neighbors Classifier.

## Results

In our study, precision is the ratio of correctly predicted falls to the total number of correctly and incorrectly predicted falls; sensitivity is the ratio of correctly predicted falls of total reported falls; and accuracy is the ratio of falls and non-falls correctly predicted by the total number of observations of falls and non-falls. The results in Tables 1 to 3 (Values within parenthesis represent the 1 standard deviation uncertainty of the calculation.) shows that none of the ML algorithms were able to discriminate non-fallers from fallers based on the low sensitivity of the different algorithms. (even when anyone presented a value close to 1, the other group presented a lower value).

**Table 1:** Precision values for different machine learning algorithms applied to sort COP data according to age, falls and fall frequency.

	Age		Falls		Fall frequency	
	Young	Old	No falls	1+ falls	Non-faller	Faller
**Logistic Regression**	0.59	0.45	0.74	0.50	0.91	1.00
**Linear Discriminant Analysis**	0.53	0.36	0.62	0.08	0.84	0.07
**K-nearest neighbors**	0.47	0.29	0.73	0..00	0.88	0.00
**Decision Tree**	0.50	0.42	0.75	0.33	0.90	0.25
**Gaussian Naive Bayes**	0.74	0.64	0.75	1.00	0.90	0.50
**C-Support Vector Classification**	0.54	0.33	0.73	0.00	1.00	0.94

**Table 2:** Sensitivity values for different machine learning algorithms applied to sort COP data according to age, falls and fall frequency.

	Age		Falls		Fall frequency	
	Young	Old	No falls	1+ falls	Non-faller	Faller
**Logistic Regression**	0.68	0.36	0.96	0.11	1.00	0.25
**Linear Discriminant Analysis**	0.53	0.36	0.54	0.11	0.55	0.25
**K-nearest neighbors**	0.47	0.29	1.00	0.00	1.00	0.00
**Decision Tree**	0.35	0.43	0.75	0.33	0.90	0.25
**Gaussian Naive Bayes**	0.74	0.64	1.00	0.11	0.50	0.25
**C-Support Vector Classification**	0.68	0.21	1.00	0.00	0.00	0.00

**Table 3:** Accuracy values of different machine learning algorithms applied to sort COP data according to age, falls and fall frequency.

	Age	Falls	Fall frequency
**Logistic Regression**	0.63(11)	0.70(80)	0.95(23)
**Linear Discriminant Analysis**	0.52(13)	0.52(12)	0.54(14)
**K-nearest neighbors**	0.52(15)	0.72(62)	0.92(1)
**Decision Tree**	0.58(13)	0.66(13)	0.84(49)
**Gaussian Naive Bayes**	0.65(12)	0.58(78)	0.71(15)
**C-Support Vector Classification**	0.64(16)	0.74(35)	0.92(1)

**Fig 1:**
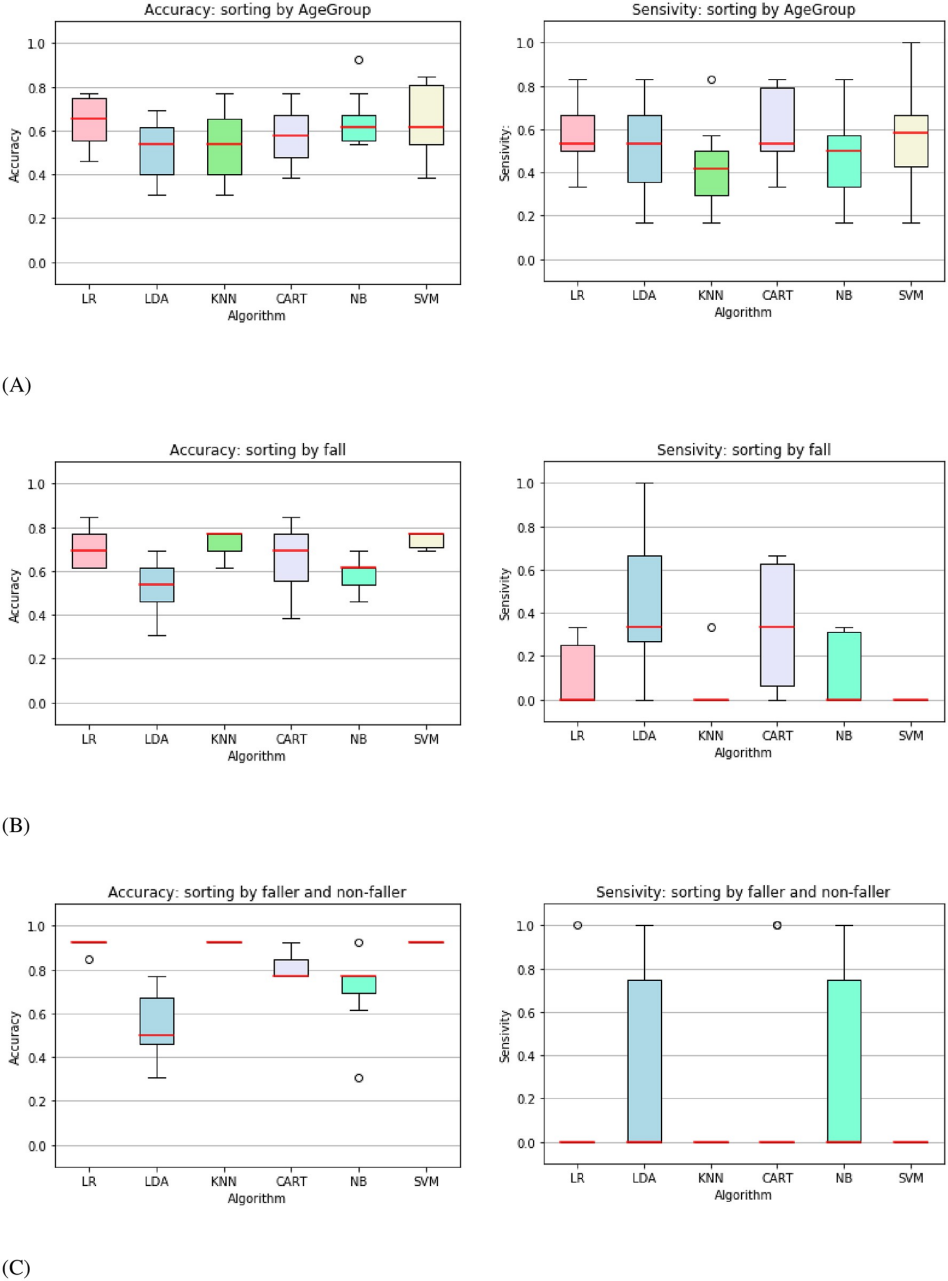
Accuracy and sensitivity values comparison between supervised learning algorithms. Logistic Regression (LR), Linear Discriminant Analysis (LDA), K-nearest neighbors (KNN), Decision Tree (CART), Gaussian Naive Bayes (NB) and C-Support Vector Classification (SVM) were applied in different sorting types in the referred human balance database: (A) Sorting by age: Young and Old; (B) Sorting by falls: No Falls and 1+ Falls; (C) Sorting by fall frequency: Faller and Non-faller.

A graphic comparison of all algorithms’ accuracy and sensitivity values can be seen in Fig 1. This data visualization shows the ML algorithms were only effective in distinguishing young from elderly.

## Discussion

This study applied six different supervisioned ML algorithms to classify 163 adults into different types of sorting, but mostly to find out if non-fallers (who had never fallen one year before data collection) and fallers (who had fallen at least once up to one year before data collection) could be sorted, with the aim of creating a tool with the ability to predict adults who are at greater risk of falling from an easily applicable test. The ML algorithms used 53 features to build these models which were not capable of classifying the participants at higher risk of falling in our sample. The ML methods had a good performance only when classifying the participants into young and old adults based on their static balance test.

Analyzing the models performance (Tables 1–3), all algorithms had good to satisfactory to good results for the three types of classification, since all accuracies were above a 60% threshold, with a maximum mean accuracy of 0.91(51) for the logistic regression regarding the separation between faller and non-faller individuals. However, low values of precision and sensitivity were observed for the fall classifications; in some cases, these values were null (LR, LDA and SVM algorithms). This means no data point was correctly labeled during the validation tests. This behavior is related to the nature of the dataset used, where the event classes (fallers and non-fallers) are unbalanced. Such classification is precisely the objective of this work. Therefore, ML methods performed well only when used to classify a patient into young and old, as can be seen in Fig 1a, based on their static balance test, failing classifications related to falls.

Although this result is disappointing from a health care and technological perspective, as in developing of an easy manner for detection of people at high risk of falling, on the other hand our results indicate a warning sign to the widespread use of standardized balance measures in the adult population to avoid falls.

The postural control research field presents a large number of independently standardized tests and measures to assess balance in adults (i.e:Activity-based Balance Level Evaluation scale, BESTest, Berg Balance Scale, Community Balance and Mobility scale), in addition to not encompassing some components that contribute considerably to the balance [42]; this variety of tests impairs the comparison of results among them and does not allow a better understanding of the relationship between performance in balance tests and the risk of falls.

There is some evidence that mediolateral sway with eyes open [33, 43] average speed of COP [44] and area of COP movement [45] have positive correlation with numbers of falls. Nonetheless, other studies, like ours (illustrated in the Fig 1b and 1c), did not find a relation between static balance test performance and risk of falls. A hypothesis raised by Buatois 2006 was that static posturographic tests were not sensitive enough for active and independent participants since the studies that found this relationship had a significantly older population or were community-dwelling elderly [32]. In their systematic review, Piirtola et al., 2006 did not find clear differences in the participants in studies whose positive association between sway parameters and falls was shown compared with studies in which such associations were not found [46].

Recently, Cabral et al., 2020 applied different ML methods in a large dataset of community-dwelling elderly fallers aged 60 to 88 years old, an age group that should increase the test sensitivity. This study found that static posturography does not improve the prediction of recurrent and single fallers [31]. We hypothesized the comparison with young people could mark greater differences between non-fallers and fallers, but the algorithms differentiated only young and old people. The principal component analysis showed the main parameters can differentiate these age groups were the COP: the range of motion and velocity in the mediolateral direction seems to be the one that contributes the most to this difference. These findings corroborate that the decrease in balance control is age-related, as shown in systematic review: in a static posturographic test with open or closed eyes, for COP displacement the differences between data in two age groups can vary 20% to 30% in anteroposterior direction and 40 to 50% in mediolateral direction; for velocity, the age differences range from 30% to 50% for both directions [47].

Our algorithms identified the age groups through the static balance parameters. In contrast, while it was not successful in differentiating participants with and without a history of falls, which may indicate that we are still unable to control or know which parameters have the greatest contribution to predicting falls occurrence.

The etiology of falls is multifactorial [48], an isolated test has low specificity in predicting fall risk. It is highly likely that static tests are not challenging enough to generate a disturbance even in participants who have some kind of balance impairment. Furthermore, there is a clear lack of specificity, since almost all falls in daily living activities occur in dynamic situations [49, 50].

A limitation of this study is that the retrospective fall incidence was based in a self-report data. The sample size analysis was not performed and the number of participants among age groups is unbalanced. The sample size is also a detrimental factor for machine learning-type analyses, which generally require a larger amount of data.

Corroborating to other studies, ours showed static posturography has no effectivity to fall risk prediction. These results suggest next studies should focus on dynamic posturography to assess the risk of falls through single tests.

## Conclusion

This study applied six different machine learning algorithms in order to test the possibility of using these methods to classify adults into non-fallers and fallers, creating a tool capable of predicting adults at greater risk of falling. However, none of the models was able to classify the participants at greatest risk of falling in this sample.

Our conclusion corroborates other works in the biomechanics field, which argue that static posturography does not have the desired effectiveness in predicting the risk of falling. This suggests that further studies should focus on dynamic posturography to assess the risk of falls through unique tests.

From the point of view of the ML tools, the computational methodology was correctly applied; the failure to obtain the expected results seems to derive only from the initial biomechanical assumptions and from the behavior of the data itself.

## Supporting information

S1 Data (XLSX)

## References

[1] Salari N, Darvishi N, Ahmadipanah M, Shohaimi S, Mohammadi M. Global prevalence of falls in the older adults: a comprehensive systematic review and meta-analysis. J Orthop Surg Res. 2022;17: 1–13. doi: 10.1186/s13018-022-03222-1 35765037 PMC9238111

[2] Wild D, Nayak USL, Isaacs B. Description, classification and prevention of falls in old people at home. Rheumatology. 1981;20: 153–159. doi: 10.1093/rheumatology/20.3.153 7280490

[3] Barry E, Galvin R, Keogh C, Horgan F, Fahey T. Is the Timed Up and Go test a useful predictor of risk of falls in community dwelling older adults: A systematic review and meta- analysis. BMC Geriatr. 2014;14: 1–14. doi: 10.1186/1471-2318-14-14 24484314 PMC3924230

[4] Singh S, S.G.K P, S.T T, C.C T, S S. Association between physiological falls risk and physical performance tests among community-dwelling older adults. Clin Interv Aging. 2015;10: 1319–1326. doi: 10.2147/CIA.S79398 26316727 PMC4541555

[5] Weinstein M, Booth J. Preventing falls in older adults: A multifactorial approach. Home Heal Care Manag Pract. 2006;19: 45–50. doi: 10.1177/1084822306292232

[6] Kim T, Park J, Heo S, Sung K, Park J. Characterizing Dynamic Walking Patterns and Detecting Falls with Wearable Sensors Using Gaussian Process Methods. Sensors (Basel). 2017;17. doi: 10.3390/s17051172 28531125 PMC5470917

[7] Kim T, Xiong S. Comparison of seven fall risk assessment tools in community-dwelling Korean older women. Ergonomics. 2017;60: 421–429. doi: 10.1080/00140139.2016.1176256 27133931

[8] Bzdok D, Altman N, Krzywinski M. Points of Significance: Statistics versus machine learning. Nat Methods. 2018;15: 233–234. doi: 10.1038/nmeth.4642 30100822 PMC6082636

[9] Chen JH, Asch SM. Machine Learning and Prediction in Medicine—Beyond the Peak of Inflated Expectations. N Engl J Med. 2017;376: 2507–2509. doi: 10.1056/NEJMp1702071 28657867 PMC5953825

[10] Rajkomar A, Dean J, Kohane I. Machine Learning in Medicine. N Engl J Med. 2019;380: 1347–1358. doi: 10.1056/NEJMra1814259 30943338

[11] Delahanty RJ, Alvarez JA, Flynn LM, Sherwin RL, Jones SS. Development and Evaluation of a Machine Learning Model for the Early Identification of Patients at Risk for Sepsis. Ann Emerg Med. 2019;73: 334–344. doi: 10.1016/j.annemergmed.2018.11.036 30661855

[12] Fleuren LM, Klausch TLT, Zwager CL, Schoonmade LJ, Guo T, Roggeveen LF, et al. Machine learning for the prediction of sepsis: a systematic review and meta-analysis of diagnostic test accuracy. Intensive Care Med. 2020;46: 383–400. doi: 10.1007/s00134-019-05872-y 31965266 PMC7067741

[13] Giannini HM, Ginestra JC, Chivers C, Draugelis M, Hanish A, Schweickert WD, et al. A Machine Learning Algorithm to Predict Severe Sepsis and Septic Shock: Development, Implementation, and Impact on Clinical Practice. Crit Care Med. 2019;47: 1485–1492. doi: 10.1097/CCM.0000000000003891 31389839 PMC8635476

[14] Tahir Manap. Parkinsons Disease Gait Classification Based on Machine Learning. 2012.

[15] Song JH, Venkatesh SS, Conant EA, Arger PH, Sehgal CM. Comparative analysis of logistic regression and artificial neural network for computer-aided diagnosis of breast masses. Acad Radiol. 2005;12: 487–495. doi: 10.1016/j.acra.2004.12.016 15831423

[16] Stylianou N, Akbarov A, Kontopantelis E, Buchan I, Dunn KW. Mortality risk prediction in burn injury: Comparison of logistic regression with machine learning approaches. Burns. 2015;41: 925–934. doi: 10.1016/j.burns.2015.03.016 25931158

[17] Hu B, Dixon PC, Jacobs JV., Dennerlein JT, Schiffman JM. Machine learning algorithms based on signals from a single wearable inertial sensor can detect surface- and age-related differences in walking. J Biomech. 2018;71: 37–42. doi: 10.1016/j.jbiomech.2018.01.005 29452755

[18] Zhang L, Ma O, Fabre JM, Wood RH, Garcia SU, Ivey KM, et al. Classification of older adults with/without a fall history using machine learning methods. Proc Annu Int Conf IEEE Eng Med Biol Soc EMBS. 2015;2015-Novem: 6760–6763. doi: 10.1109/EMBC.2015.7319945 26737845

[19] Lötters JC, Schipper J, Veltink PH, Olthuis W, Bergveld P, Wu YY, et al. Fall detection algorithms for real-world falls harvested from lumbar sensors in the elderly population: A machine learning approach BT—38th Annual International Conference of the IEEE Engineering in Medicine and Biology Society, EMBC 2016, August 16, 2. Sensors (Switzerland). 2018;18: 1–14. doi: 10.3390/s19071644 28269098

[20] Minvielle L, Atiq M, Serra R, Mougeot M, Vayatis N. Fall detection using smart floor sensor and supervised learning. Proc Annu Int Conf IEEE Eng Med Biol Soc EMBS. 2017; 3445–3448. doi: 10.1109/EMBC.2017.8037597 29060638

[21] Bourke AK, Klenk J, Schwickert L, Aminian K, Ihlen EAF, Mellone S, et al. Fall detection algorithms for real-world falls harvested from lumbar sensors in the elderly population: A machine learning approach. Proc Annu Int Conf IEEE Eng Med Biol Soc EMBS. 2016;2016-Octob: 3712–3715. doi: 10.1109/EMBC.2016.7591534 28269098

[22] Aziz O, Musngi M, Park EJ, Mori G, Robinovitch SN. A comparison of accuracy of fall detection algorithms (threshold-based vs. machine learning) using waist-mounted tri-axial accelerometer signals from a comprehensive set of falls and non-fall trials. Med Biol Eng Comput. 2017;55: 45–55. doi: 10.1007/s11517-016-1504-y 27106749

[23] Özdemir AT, Barshan B. Detecting falls with wearable sensors using machine learning techniques. Sensors (Switzerland). 2014;14: 10691–10708. doi: 10.3390/s140610691 24945676 PMC4118339

[24] Wang L, Xue Z, Ezeana CF, Puppala M, Chen S, Danforth RL, et al. Preventing inpatient falls with injuries using integrative machine learning prediction: a cohort study. npj Digit Med. 2019;2: 1–7. doi: 10.1038/s41746-019-0200-3 31872067 PMC6908660

[25] Young AJ, Hare A, Subramanian M, Weaver JL, Kaufman E, Sims C. Using Machine Learning to Make Predictions in Patients Who Fall. J Surg Res. 2021;257: 118–127. doi: 10.1016/j.jss.2020.07.047 32823009

[26] Lee SK, Ahn J, Shin JH, Lee JY. Application of machine learning methods in nursing home research. Int J Environ Res Public Health. 2020;17: 1–15. doi: 10.3390/ijerph17176234 32867250 PMC7503291

[27] Patterson BW, Engstrom CJ, Sah V, Smith MA, Mendonça EA, Pulia MS, et al. Training and Interpreting Machine Learning Algorithms to Evaluate Fall Risk After Emergency Department Visits. Med Care. 2019;57: 560–566. doi: 10.1097/MLR.0000000000001140 31157707 PMC6590914

[28] Gillain S, Boutaayamou M, Schwartz C, Brüls O, Bruyère O, Croisier JL, et al. Using supervised learning machine algorithm to identify future fallers based on gait patterns: A two-year longitudinal study. Exp Gerontol. 2019;127: 110730. doi: 10.1016/j.exger.2019.110730 31520696

[29] Martinez M, De Leon PL. Falls Risk Classification of Older Adults Using Deep Neural Networks and Transfer Learning. IEEE J Biomed Heal Informatics. 2020;24: 144–150. doi: 10.1109/JBHI.2019.2906499 30932855

[30] Qiu H, Rehman RZU, Yu X, Xiong S. Application of Wearable Inertial Sensors and A New Test Battery for Distinguishing Retrospective Fallers from Non-fallers among Community-dwelling Older People. Sci Rep. 2018;8: 1–10. doi: 10.1038/s41598-018-34671-6 30397282 PMC6218502

[31] Cabral K de N, Brech GC, Alonso AC, Soares AT, Opaleye DC, Greve JMD, et al. Posturographic measures did not improve the predictive power to identify recurrent falls in community-dwelling elderly fallers. Clinics. 2020;75. doi: 10.6061/clinics/2020/e1409 32267394 PMC7100920

[32] Buatois S, Gueguen R, Gauchard GC, Benetos A, Perrin PP. Posturography and risk of recurrent falls in healthy non-institutionalized persons aged over 65. Gerontology. 2006;52: 345–352. doi: 10.1159/000094983 16905886

[33] Howcroft J, Lemaire ED, Kofman J, McIlroy WE. Elderly fall risk prediction using static posturography. PLoS One. 2017;12. doi: 10.1371/journal.pone.0172398 28222191 PMC5319679

[34] Santos DA, Duarte M. A public data set of human balance evaluations. PeerJ. 2016. doi: 10.7717/peerj.2648 27833813 PMC5101613

[35] Scikit-learn. Logistic regression. 2022. https://scikit-learn.org/stable/modules/linear_model.html#logistic-regression.

[36] Scikit-learn. Linear and Quadratic Discriminant Analysis. 2022. https://scikit-learn.org/stable/modules/lda_qda.html.

[37] Mitchell TM. Machine Learning. McGraw-Hill. 1997; 414.

[38] Scikit-learn. Decision Trees, 2022. https://scikit-learn.org/stable/modules/decision_tree.html.

[39] Scikit-learn. Gaussian Naive Bayes. 2022. https://scikit-learn.org/stable/modules/naive_bayes.html

[40] Handelman GS, Kok HK, Chandra RV., Razavi AH, Lee MJ, Asadi H. eDoctor: machine learning and the future of medicine. J Intern Med. 2018;284: 603–619. doi: 10.1111/joim.12822 30102808

[41] Brownlee J. Your First Machine Learning Project in Python Step-By-Step. 2019. Available: https://machinelearningmastery.com/machine-learning-in-python-step-by-step/.

[42] Sibley KM, Beauchamp MK, Van Ooteghem K, Straus SE, Jaglal SB. Using the systems framework for postural control to analyze the components of balance evaluated in standardized balance measures: A scoping review. Arch Phys Med Rehabil. 2015;96: 122–132.e29. doi: 10.1016/j.apmr.2014.06.021 25073007

[43] Stel VS, Smit JH, Pluijm SMF, Lips P. Balance and mobility performance as treatable risk factors for recurrent falling in older persons. J Clin Epidemiol. 2003;56: 659–668. doi: 10.1016/s0895-4356(03)00082-9 12921935

[44] Maki BE, Holliday PJ, Fernie GR. Aging and Postural Control. J Am Geriatr Soc. 1990. doi: 10.1111/j.1532-5415.1990.tb01588.x 2295764

[45] Thapa PB, Gideon P, Brockman KG, Fought RL, Ray WA. Clinical and Biomechanical Measures of Balance as Fall Predictors in Ambulatory Nursing Home Residents. J Gerontol Med Sci. 1996. Available: https://academic.oup.com/biomedgerontology/article/51A/5/M239/578807 doi: 10.1093/gerona/51a.5.m239 8808996

[46] Piirtola M, Era P. Force platform measurements as predictors of falls among older people—A review. Gerontology. 2006. doi: 10.1159/000089820 16439819

[47] Roman-Liu D Age-related changes in the range and velocity of postural sway. Arch Gerontol Geriatr. 2018;77: 68–80. doi: 10.1016/j.archger.2018.04.007 29684741

[48] Close JCT, Lord SR. Fall assessment in older people. BMJ. 2011;343: 1–6. doi: 10.1136/bmj.d5153 21917828

[49] Rezende AAB, Silva L e, Cardoso FB, Beresford H. Fear among the elderly of suffering recurring falls: the gait as a determining factor of functional independence. Acta Fisiátrica. 2010;17: 117–121. doi: 10.11606/issn.2317-0190.v17i3a103353

[50] De Vriendt P, Gorus E, Cornelis E, Velghe A, Petrovic M, Mets T. The process of decline in advanced activities of daily living: A qualitative explorative study in mild cognitive impairment. Int Psychogeriatrics. 2012;24: 974–986. doi: 10.1017/S1041610211002766 22301014

