# Correction: Fall prediction in a quiet standing balance test via machine learning: Is it possible?

**DOI:** 10.1371/journal.pone.0322963

**Published:** 2025-04-22

**Authors:** Juliana Pennone, Natasha Fioretto Aguero, Daniel Marczuk Martini, Luis Mochizuki, Alexandre Alarcon do Passo Suaide

The Funding statement for this article is incorrect. The correct Funding statement is as follows: This work was partially funded by São Paulo Research Agency (FAPESP), grant number 2020/04867-2.
